# Patient-related factors influencing the effectiveness and safety of Janus Kinase inhibitors in rheumatoid arthritis: a real-world study

**DOI:** 10.1038/s41598-023-50379-8

**Published:** 2024-01-02

**Authors:** Cristina Martinez-Molina, Ignasi Gich, Cesar Diaz-Torné, Hye S. Park, Anna Feliu, Silvia Vidal, Hèctor Corominas

**Affiliations:** 1 Department of Pharmacy, Hospital de la Santa Creu i Sant Pau, Barcelona, Spain; 2 Department of Medicine, Universitat Autònoma de Barcelona (UAB), Barcelona, Spain; 3 CIBER Epidemiología y Salud Pública (CIBERESP), Instituto de Salud Carlos III, Madrid, Spain; 4 Department of Clinical Epidemiology and Public Health, Hospital de la Santa Creu i Sant Pau, Barcelona, Spain; 5 Department of Rheumatology and Systemic Autoimmune Diseases, Hospital de la Santa Creu i Sant Pau, 89 Sant Quinti Street, 5th Floor, 08041 Barcelona, Spain; 6 Group of Immunology-Inflammatory Diseases, Sant Pau Biomedical Research Institute (IIB Sant Pau), Barcelona, Spain; 7 Department of Rheumatology and Systemic Autoimmune Diseases, Department of Medicine, Universitat Autònoma de Barcelona (UAB), Barcelona, Spain

**Keywords:** Rheumatic diseases, Translational research, Autoimmune diseases, Inflammatory diseases

## Abstract

In real-world scenarios, Janus Kinase (JAK) inhibitors are often offered to "difficult-to-treat" rheumatoid arthritis patients, quite different from those included in randomized controlled trials. Our study aimed to evaluate the influence of patient-related factors on the effectiveness and safety of JAK inhibitors in real-world clinical practice. This observational retrospective study involved rheumatoid arthritis patients who received treatment with either tofacitinib, baricitinib, upadacitinib, or filgotinib. At 12 months of treatment, reasons for and rates of JAK inhibitor treatment discontinuation were examined. Treatment retentions were analyzed through Cox proportional hazard regression models and Kaplan–Meier estimates. Patient-related factors that could influence treatment retention were evaluated for the discontinuation reasons of lack of effectiveness and adverse events. At 12 months of treatment, discontinuation rates for 189 JAK inhibitor treatments were: lack of effectiveness (24.3%), adverse events (20.6%), and other reasons (3.7%). The remaining 51.4% represents the treatment continuation rate. No patient-related factors evaluated had an influence on treatment discontinuation due to lack of effectiveness. Ae significantly increased the risk of treatment discontinuation due to adverse events (p = 0.030). In terms of age, at 12 month of treatment, discontinuation rates due to adverse events were: < 65 years, 14.4% vs. 65 years or older, 26.3% (p = 0.019). Rheumatoid arthritis patients aged 65 years or older showed an increased risk of JAK inhibitor treatment discontinuation due to adverse events. Factors not related to treatment discontinuation were: sex, rheumatoid arthritis disease duration, rheumatoid arthritis disease activity, seropositivity for rheumatoid factor, seropositivity for anti-cyclic citrullinated peptides, number of prior biologic treatments, number of prior JAK inhibitor treatments, concomitant use of glucocorticoids, and concomitant use of conventional synthetic disease-modifying antirheumatic drugs.

## Introduction

Rheumatoid arthritis (RA) is a chronic inflammatory autoimmune disorder that primarily affects women and typically presents during the sixth decade of life^1^. The pathophysiology of RA is characterized by chronic inflammation of the synovial membrane, leading to the progressive destruction of articular cartilage and marginal bone^2^. The primary goal for the treatment of patients with RA is to control the inflammation, aiming to prevent irreversible damage^2^. The recommendations from the European League Against Rheumatism (EULAR), in accordance with the treatment guidelines of the American College of Rheumatology (ACR), emphasize the importance of initiating treatment at the time of diagnosis^3,4^. First-line treatment typically consists of administering conventional synthetic (cs) Disease-Modifying Antirheumatic Drugs (DMARDs), mainly methotrexate (MTX), either as monotherapy or in combination with short-term and low dose glucocorticoids (GC)^3,4^. If this treatment fails, and remission or low disease activity is not achieved within a 6-month period, a second-line treatment approach should be pursued. This approach involves add-on therapy with a biologic DMARD (bDMARD) or, assuming risk assessment, a Janus Kinase (JAK) inhibitor^3^. If a JAK inhibitor fails, it turns out to consider again other JAK inhibitor or a bDMARD to end the loop^3^.

Several orally available JAK inhibitors have been developed for the management of RA. Tofacitinib (TOF), baricitinib (BAR), upadacitinib (UPA), and filgotinib (FIL) have demonstrated significant long-term efficacy and safety across diverse randomized controlled trials (RCTs)^5–24^, and currently, they constitute the four approved small molecules for RA treatment in Europe. However, RCTs commonly involve a smaller, well-selected study population that is closely monitored under predefined conditions and time intervals^25,26^. Furthermore, patients commonly enrolled in RCTs differ from those typically encountered in real-world settings^26^.

In real-world clinical practice, JAK inhibitors are often offered to patients who experienced multiple failures to bDMARDs and, increasingly, to other JAK inhibitors; who exhibit active/progressive disease activity; and whose RA management is perceived as problematic. Those patients are commonly referred to as "difficult-to-treat" RA patients^27^. Therefore, real-world evidence (RWE) studies, whether prospective or retrospective, can significantly complement the information obtained from RCTs, providing valuable insights that enhance healthcare decision-making^25,26^.

The main aim of the present study was to evaluate the influence of patient-related factors on the retention of JAK inhibitor treatment in RA patients within real-world scenarios. Within clinical practice, treatment retention serves as a composite metric that indirectly indicates the effectiveness and safety of a given treatment.

## Methods

### Study design and patient population

This is an observational, retrospective, single-center study that involved real-world patients who fulfilled the 2010 ACR-EULAR classification criteria for RA^28^.

In a tertiary-care university hospital from Spain, three rheumatologists, following clinical guidelines based on the EULAR recommendations^3^, attended to patients with RA. Patients who received treatment with either TOF, BAR, UPA, or FIL between September 2017 and May 2023, and who had comprehensive data regarding treatment initiation, potential discontinuation, and reasons for discontinuation, were eligible for inclusion in this study. All patients included were individually informed about the study and were given the option to decline the extraction of data from their electronic medical records. Data were retrospectively collected from patients’ records between March 2022 and May 2023.

### Assessments

The retention of treatment was defined as the time interval between treatment initiation and definitive treatment discontinuation. The reasons for discontinuation were classified into three primary categories, as outlined below: (1) lack of effectiveness (including primary and secondary failure), (2) adverse events, and (3) other reasons. Physicians were restricted to select a single reason for discontinuation.

The potential predictive factors for JAK inhibitor retention included socio-demographic information (age, sex), RA anamnesis (RA disease duration), RA disease activity (measured using the Clinical Disease Activity Index (CDAI)), RA seropositivity (Rheumatoid Factor (RF), anti-Cyclic Citrullinated Peptides (anti-CCP)), number of prior RA treatments (previous bDMARDs, previous JAK inhibitors), and the presence or absence of concomitant RA treatments (GC, csDMARDs).

While RA can present at any age, its prevalence notably escalates with advanced age, with a substantial portion of patients experiencing initial symptoms after 60 years of old^1^. Accordingly, patients were categorized by age: young (< 65 years) and old (65 years or older). The CDAI scale was deemed appropriate for measuring disease activity. The CDAI does not incorporate acute phase reactants, making it more applicable for assessing disease activity, particularly when drugs have significantly influenced these inflammation biomarkers.

### Statistical analyses

Differences in baseline patient characteristics among the JAK inhibitor groups (TOF, BAR, UPA, FIL) were evaluated using the Kruskal–Wallis test or the analysis of variance (for ordinal or quantitative variables) and the Fisher's exact test (for categorical variables).

Treatment retention was examined through Cox proportional hazard regression models and Kaplan–Meier estimates. Cox proportional hazard regression models (bivariate and multivariate) were applied to analyze the potential predictive patient-related factors described previously, which were present at the initiation of JAK inhibitor treatment. These potential predictive factors could influence the treatment retention for the discontinuation reasons of (1) lack of effectiveness and (2) adverse events, while excluding (3) other reasons. Covariates with a *P* value < 0.1 from the bivariate analysis were included in the multivariate analysis. Kaplan–Meier estimates, for the specified discontinuation reasons, were employed to evaluate the survival curves of treatment retention based on the potential predictive factors, with the log-rank test used for comparison. At the 12-month mark of JAK inhibitor treatment, reasons for and rates of treatment discontinuation were examined.

The statistical analyses were performed using STATA software version 15. A *P* value of < 0.05 was considered statistically significant.

### Ethics approval and consent to participate

This research study was conducted retrospectively from data obtained for clinical purposes. We consulted extensively with the Research Ethics Committee of Hospital de la Santa Creu i Sant Pau who determined that our study did not need ethical approval. An official waiver of ethical approval was granted from the Research Ethics Committee of Hospital de la Santa Creu i Sant Pau.

All procedures involved in the present study were in accordance with the 1964 Helsinki declaration and its later amendments or comparable ethical standards. Informed consent was obtained from patient included in the study.

## Results

### Study population

Between September 2017 and May 2023, a total of 189 JAK inhibitor treatments were identified, corresponding to 123 RA patients. Demographic and clinical characteristics of the included patients at the initiation of treatment are summarized in Table 1.

**Table 1:** Demographic and clinical characteristics at JAK inhibitor treatment initiation.

Parameters	TOF (n = 66)	BAR (n = 93)	UPA (n = 14)	FIL (n = 16)	*p* value	Total (n = 189)
Age (years), mean ± SD	61.7 ± 13.2	63.4 ± 12.9	63.7 ± 12.9	63.2 ± 12.6	0.994	62.8 ± 12.9
Sex (female), n (%)	55 (83.3)	84 (90.3)	11 (78.6)	13 (81.3)	0.322	163 (86.2)
BMI (weight(kg)/height(m^2^)), mean ± SD	26.5 ± 4.6	27.7 ± 5	27.5 ± 4.3	26.9 ± 5.5	0.742	27.2 ± 4.8
RA disease duration (years), median (IQR)	13 (5–22)	13 (5–23)	14.5 (11–21)	19 (15.5–27.5)	0.333	14 (6–23)
RF (positivity), n (%)	37 (56.1)	59 (64.1)	10 (71.4)	9 (56.3)	0.620	115 (61.2)
Anti-CCP (positivity), n (%)	49 (74.2)	71 (76.3)	11 (78.6)	10 (62.5)	0.674	141 (74.6)
DAS28-ESR, median (IQR)	5.2 (3.9–6)	5.1 (4.1–5.9)	5.3 (4–5.7)	5.4 (4.4–5.6)	0.995	5.1 (4.2–5.9)
DAS28-CRP, median (IQR)	4.7 (3.9–5.3)	4.5 (4–5.3)	4.7 (4.4–4.9)	4.5 (4.1–4.9)	0.788	4.6 (3.9–5.2)
CDAI, median (IQR)	23 (18–32.5)	23 (16.5–32)	23.5 (21–28)	26 (17–29)	0.996	23 (18–31)
SDAI, median (IQR)	23.2 (15.1–31.3)	22.8 (16.1–31.7)	24.2 (22.6–28.8)	26.1 (18.3–30.1)	0.908	23.2 (16.2–31.1)
Concomitant GC, n (%)	42 (63.6)	50 (53.8)	5 (35.7)	10 (62.5)	0.231	107 (56.6)
PDN dose equivalent (mg/day), median (IQR)	5 (5–10)	5 (5–6)	7.5 (5–10)	5 (5–10)	0.282	5 (5–8)
Concomitant csDMARDs, n (%)	22 (33.3)	25 (26.9)	1 (7.1)	2 (12.5)	0.125	50 (26.5)
MTX use, n (%)	12 (18.2)	16 (17.2)	1 (7.1)	1 (6.3)	0.635	30 (15.9)
LEF use, n (%)	3 (4.6)	3 (3.2)	0 (0)	0 (0)	0.893	6 (3.2)
SSZ use, n (%)	3 (4.6)	4 (4.3)	0 (0)	1 (6.3)	0.932	8 (4.2)
Previous bDMARDs (number), median (IQR)	2 (1–4)	3 (1–4)	3 (3–7)	3 (2–6.5)	0.104	3 (1–5)
Prior TNFi use, n (%)	44 (66.7)	67 (72)	13 (92.9)	14 (87.5)	0.121	138 (73)
Prior IL6i use, n (%)	36 (54.6)	51 (54.8)	9 (64.3)	11 (68.8)	0.702	107 (56.6)
Prior CD80/86i use, n (%)	30 (45.5)	38 (40.9)	7 (50)	10 (62.5)	0.428	85 (45)
Prior CD20i use, n (%)	17 (25.8)	21 (22.6)	4 (28.6)	6 (37.5)	0.593	48 (25.4)
Previous JAK inhibitors (number), median (IQR)	0 (0–0)	0 (0–1)	1 (1–2)	1 (0.5–2)	< 0.001	0 (0–1)
Prior TOF use, n (%)	0 (0)	24 (25.8)	10 (71.4)	9 (56.3)	< 0.001	43 (22.8)
Prior BAR use, n (%)	13 (19.7)	1 (1.1)	9 (64.3)	10 (62.5)	< 0.001	33 (17.5)
Prior UPA use, n (%)	0 (0)	0 (0)	0 (0)	3 (18.8)	0.001	3 (1.6)
Prior FIL use, n (%)	0 (0)	0 (0)	1 (7.1)	0 (0)	0.074	1 (0.5)

*TOF* tofacitinib, *BAR* baricitinib, *UPA* upadacitinib, *FIL* filgotinib, *BMI* body mass index, *RA* rheumatoid arthritis, *RF* rheumatoid factor, *anti-CCP* anti-Cyclic Citrullinated Peptides, *DAS28-ESR* disease activity score 28‐joint count using erythrocyte sedimentation rate, *DAS28-CRP* disease activity score 28‐joint count using C‐reactive protein, *CDAI* clinical disease activity index, *SDAI* simplified disease activity index, *GC* glucocorticoids, *PDN* prednisone, *csDMARDs* conventional synthetic disease-modifying antirheumatic drugs, *MTX* methotrexate, *LEF* leflunomide, *SSZ* sulfasalazine, *bDMARDs* biologic disease-modifying antirheumatic drugs, *TNFi* tumor necrosis factor inhibitor, *IL6i* interleukin 6 inhibitor, *CD80/86i* cluster of differentiation 80/86 inhibitor, *CD20i* cluster of differentiation 20 inhibitor, *JAK* Janus Kinase.

Differences between the groups were evaluated utilizing the Kruskal–Wallis test, the analysis of variance, or the Fisher's exact test. *P* < 0.05.

### Reasons and rates of treatment discontinuation

After 12 months of treatment, JAK inhibitor discontinuation rates due to the corresponding reasons were as follows: lack of effectiveness (24.3%), adverse events (20.6%), and other reasons (3.7%). The remaining 51.4% represents the treatment continuation rate.

With regard to JAK inhibitor treatment discontinuation due to lack of effectiveness (Table 2), bivariate Cox regression analyses suggested that a greater number of previous bDMARDs treatments, a higher disease activity according to the CDAI scale, the concomitant use of GC, and an increased number of previous JAK inhibitors treatments, could represent potential patient-related factors associated with the prognostic risk of treatment discontinuation. However, following multiple imputation, no independent risk factors were found to significantly impact the JAK inhibitors' effectiveness to lead to treatment discontinuation.

**Table 2:** Cox proportional hazard analysis for risk factors of JAK inhibitor treatment discontinuation due to lack of effectiveness.

Covariate	Bivariate analysis		Multivariate analysis	
	HR [95% CI]	*p* value	HR [95% CI]	*p* value
Previous bDMARDs (number)	1.18 [1.07–1.31]	0.001	1.11 [0.97–1.26]	0.125
CDAI	1.03 [1.00–1.05]	0.023	1.02 [0.99–1.05]	0.121
Previous JAK inhibitors (number)	1.48 [1.05–2.07]	0.025	1.20 [0.79–1.83]	0.381
Concomitant GC	1.68 [0.96–2.94]	0.072	1.43 [0.80–2.56]	0.223
Concomitant csDMARDs	0.64 [0.34–1.19]	0.158		
RA disease duration (years)	0.98 [0.96–1.01]	0.163		
RF (positivity)	0.71 [0.42–1.21]	0.210		
Age (years)	0.81 [0.48–1.38]	0.437		
Anti-CCP (positivity)	0.89 [0.48–1.66]	0.720		
Sex (female)	1.08 [0.53–2.21]	0.826		

*HR* hazard ratio, *CI* confidence interval, *bDMARDs* biologic disease-modifying antirheumatic drugs, *CDAI* clinical disease activity index, *JAK* Janus Kinase, *GC* glucocorticoids, *csDMARDs* conventional synthetic disease-modifying antirheumatic drugs, *RA* rheumatoid arthritis, *RF* rheumatoid factor, *anti-CCP* anti-cyclic citrullinated peptides.

All covariates that were statistically significant (*P* < 0.05) or exhibited borderline significance (*P* < 0.1 and > 0.05) in the bivariate analysis were included in the multivariate analysis.

Concerning JAK inhibitor cessation due to adverse events (Table 3), bivariate Cox regression analyses showed that age greater than 65 years and female sex could be potential patient-related factors associated with an increased prognostic risk of treatment discontinuation. In contrast, anti-CCP positivity and RF positivity could be associated with a potential protective prognostic effect against treatment discontinuation due to safety concerns. The results of the multivariate Cox regression analysis indicated that an age greater than 65 years seems to significantly increase the risk of JAK inhibitor treatment discontinuation due to adverse events (HR: 1.98; p = 0.030).

**Table 3:** Cox proportional hazard analysis for risk factors of JAK inhibitor treatment discontinuation due to adverse events.

Covariate	Bivariate analysis		Multivariate analysis	
	HR [95% CI]	*p* value	HR [95% CI]	*p* value
Age (years)	2.05 [1.11–3.80]	0.022	1.98 [1.07–3.67]	0.030
Anti-CCP (positivity)	0.53 [0.29–0.96]	0.036	0.61 [0.28–1.33]	0.212
Sex (female)	2.79 [0.87–9.01]	0.085	2.44 [0.75–7.88]	0.137
RF (positivity)	0.61 [0.34–1.08]	0.090	0.84 [0.40–1.79]	0.658
Previous bDMARDs (number)	1.09 [0.97–1.22]	0.149		
Concomitant csDMARDs	0.62 [0.31–1.25]	0.181		
CDAI	1.01 [0.98–1.04]	0.549		
Previous JAK inhibitors (number)	1.12 [0.73–1.72]	0.592		
RA disease duration (years)	1.01 [0.98–1.03]	0.656		
Concomitant GC	1.12 [0.63–2.01]	0.702		

*HR* hazard ratio, *CI* confidence interval, *anti-CCP* anti-cyclic citrullinated peptides, *RF* rheumatoid factor, *bDMARDs* biologic disease-modifying antirheumatic drugs, *csDMARDs* conventional synthetic disease-modifying antirheumatic drugs, *CDAI* clinical disease activity index, *JAK* Janus Kinase, *RA* rheumatoid arthritis, *GC* glucocorticoids.

All covariates that were statistically significant (*P* < 0.05) or exhibited borderline significance (*P* < 0.1 and > 0.05) in the bivariate analysis were included in the multivariate analysis.

**Figure 1:**
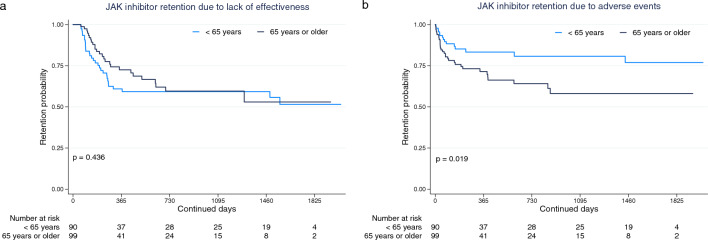
Treatment retention of JAK inhibitors by age. Treatment retention between young (< 65 years) and old (65 years or older) patients, due to (**a**) lack of effectiveness and (**b**) adverse events.

In terms of age, at 12 months of treatment, no differences were observed in the discontinuation rates due to lack of effectiveness (p = 0.436; Fig. 1a), while there were significant differences noted for those related to adverse events, which were: young, 14.4% vs. old, 26.3% (p = 0.019; Fig. 1b).

## Discussion

This study assessed patient-related factors associated with the retention of JAK inhibitor treatment, thereby investigating how patient characteristics impact the effectiveness and safety of these small molecules. Based on the available literature, there is limited evidence addressing these issues within real-world conditions. Ebina et al. explored Asian RA patients treated in accordance with Japanese guidelines using either TOF or BAR^29^. To the best of our knowledge, this present study is the first to include treatments with TOF, BAR, UPA, and FIL, the four JAK inhibitors currently approved for RA management in Europe.

Regarding the impact of age, both young and old RA patients displayed similar efficacy and effectiveness when treated with JAK inhibitors^29–31^. However, in patients aged 65 years or older, an association was observed between TOF treatment and an increased risk of cardiovascular events and malignancies when compared to Tumor Necrosis Factor inhibitor (TNFi) treatment^32^. Consequently, due to potential shared effects within the drug class, in accordance with the European Medicines Agency (EMA)^33^ and the EULAR^3^, careful consideration should be given to individuals aged over 65 years when considering the prescription of a JAK inhibitor. Consistently with this approach, in our current study, age was not found to be related to treatment discontinuation due to lack of effectiveness, but it was significantly associated with treatment discontinuation due to adverse events (HR: 1.98; p = 0.030). Regarding adverse events, the discontinuation rates at 12 months of treatment were as follows: young, 14.4% vs. old, 26.3% (p = 0.019; Fig. 1b).

In terms of sex, there is currently a lack of substantial evidence concerning its potential impact on the retention of JAK inhibitor treatment^29,34^. Our study findings suggest that being female or male does not significantly influence the effectiveness and safety of a JAK inhibitor treatment.

With regard to RA seropositivity, a post-hoc analysis of TOF treatment indicated that the treatment outcome is not significantly affected by the positivity or negativity of anti-CCP or RF^34^. In a recent study, seropositivity (anti-CCP or RF) was found to have no influence on JAK inhibitor treatment retention^34^. In line with both, the existing literature^34,35^ and our study' results, neither anti-CCP nor RF were found to have significant effects on treatment retention.

Concerning RA disease activity, when poor prognostic factors are present and patients experience moderate to severe RA activity despite the initial csDMARDs strategy, treatment with a JAK inhibitor may be considered. At 6 months of treatment, dose reduction or interval adjustment can be safety implemented with any JAK inhibitor if clinical remission, or at least low disease activity, is achieved^3^. At baseline, the patients of our study exhibited severe or, at least moderate RA activity, according with the guideline recommendations. These baseline RA disease activity values did not significantly impact the effectiveness and safety of the JAK inhibitor treatment in our study.

With respect to RA disease duration, published literature suggests that it should not be considered a factor influencing treatment retention^29,34^. Similarly, our present study did not identify any significant association between RA disease duration and discontinuation of treatment due to lack of effectiveness or adverse events.

Regarding the number of prior bDMARDs treatments, recent evidence indicates that it does not have a significant impact on JAK inhibitor treatment retention^29,34^. Similarly, in our study, comparable JAK inhibitor treatment retentions were observed regardless of the number of previous bDMARDs used. It is worth nothing that specific mechanisms of action of prior bDMARDs might suggest an increased risk of JAK inhibitor treatment discontinuation due to lack of effectiveness, such as, interleukin (IL)-6 inhibition^29^.

Another consideration relates to the number of prior JAK inhibitor treatments. The strategy of subsequent JAK inhibitor treatments, referred to as the JAK inhibitor cycling strategy, has shown to be effective and safe as an eligible option following the failure of a prior JAK inhibitor in terms of lack of effectiveness or adverse events^36^. The number of prior JAK inhibitor treatments does not seem to impact subsequent JAK inhibitor treatment retentions, as supported by both the existing literature^29^ and the findings of our study.

In reference to the presence or absence of concomitant GC, when initiating a JAK inhibitor treatment or making changes in concomitant csDMARDs, it is recommended by both the ACR guidelines^4^ and EULAR recommendations^3^ to consider short-term GC in different dose regimens and routes of administration. However, it is crucial to gradually taper and discontinue GC as soon as it is clinically feasible due to the potential risk of adverse events^3,4^. Doses exceeding 7.5 mg/day of oral prednisone (PDN) equivalent were identified as a risk factor for serious infections in TOF treatment^37^. In our present study, 56.6% of the patients were receiving GC at the initiation of JAK inhibitor treatment, with a median (IQR) PDN dose equivalent of 5 (5–8) mg. At baseline, in JAK inhibitor treatment, the presence or absence of concomitant GC at low doses (≤ 7.5 mg/day PDN equivalent^3^) is not related to the lack of effectiveness or the occurrence of adverse events, as evidenced by both the published literature^34^ and the findings of our study.

No compelling evidence exists regarding the monotherapy of JAK inhibitor compared to the combination therapy with csDMARDs^27,34^. According to the EULAR recommendations^3^, it is advocated to continue MTX (or other csDMARDs) when planning treatment with a JAK inhibitor, after assessing the associated risks. The MTX dose can be reduced to convey the added benefit of combination vs. monotherapy while, mitigating the risk of adverse events^3^. In our study, 15.9% of patients continued MTX upon JAK inhibitor initiation (Table 1). Based on our findings, no significant differences were observed between the presence or absence of concomitant csDMARDs regarding the risk of JAK inhibitor treatment discontinuation due to lack of effectiveness nor adverse events. These results are consistent with recent literature^29^.

There are limitations to the present study that inevitably influence the interpretation of the results obtained. Firstly, when extending the findings of this study to the broader population, it is crucial to take into account both the population size and the fact that the study was performed exclusively at a single healthcare center. However, it is worth mentioning that the results obtained in that study align with the previous existing evidence. The other limitation stems from the uneven distribution of JAK inhibitor treatment groups, which reflects real-world clinical practice and represents a common limitation in observational studies. Given that UPA and FIL are the most recently approved JAK inhibitors for RA, the majority of patients were treated with TOF or BAR. Due to limited statistical power, the assessment of factors influencing treatment retention based on the type of JAK inhibitor was not feasible. There is a lack of head-to-head randomized clinical trials comparing these small molecules^38^. Future research should aim to determine potential differences among various types of JAK inhibitors.

The main strength of our study resides in the inclusion of RA patients being treated on real life, examining factors that could impact the effectiveness and safety of the JAK inhibitor treatment. This is particularly significant for "difficult-to-treat" RA patients who might not be included in RCTs.

In summary, age (65 years or older) was significantly linked to an increased risk of the treatment discontinuation of JAK inhibitors due to adverse events. Patient-related factors not associated with treatment discontinuation were as follows: sex, RA disease duration, RA disease activity, seropositivity for RF, seropositivity for anti-CCP, number of prior bDMARD treatments, number of prior JAK inhibitor treatments, concomitant use of GC, and concomitant use of csDMARDs. These findings can significantly complement the information obtained from randomized controlled trials, providing valuable insights that enhance healthcare decision-making.

## Data Availability

The datasets used and/or analyzed during the current study are available from the corresponding author on reasonable request.

## Abbreviations

ACR: American college of rheumatology
anti-CCP: Anti-cyclic citrullinated peptides
BAR: Baricitinib
bDMARD: Biologic disease-modifying antirheumatic drug
BMI: Body mass index
CD20i: Cluster of differentiation 20 inhibitor
CD80/86i: Cluster of differentiation 80/86 inhibitor
CDAI: Clinical disease activity index
CI: Confidence interval
csDMARD: Conventional synthetic disease-modifying antirheumatic drug
DAS28-CRP: Disease activity score 28‐joint count using C‐reactive protein
DAS28-ESR: Disease activity score 28‐joint count using erythrocyte sedimentation rate
DMARD: Disease-modifying antirheumatic drug
EMA: European medicines agency
EULAR: European league against rheumatism
FIL: Filgotinib
GC: Glucocorticoids
HR: Hazard ratio
IL: Interleukin
IL6i: Interleukin 6 inhibitor
JAK: Janus Kinase
LEF: Leflunomide
MTX: Methotrexate
PDN: Prednisone
RA: Rheumatoid arthritis
RCTs: Randomized controlled trials
RF: Rheumatoid factor
SDAI: Simplified disease activity index
SSZ: Sulfasalazine
TNFi: Tumor necrosis factor inhibitor
TOF: Tofacitinib
UPA: Upadacitinib
